# Gestational Diabetes and the Gut Microbiota: Fibre and Polyphenol Supplementation as a Therapeutic Strategy

**DOI:** 10.3390/microorganisms12040633

**Published:** 2024-03-22

**Authors:** Carmen Pheiffer, Sylvia Riedel, Stephanie Dias, Sumaiya Adam

**Affiliations:** 1 Biomedical Research and Innovation Platform (BRIP), South African Medical Research Council, Tygerberg, Cape Town 7505, South Africa; sylvia.riedel@mrc.ac.za (S.R.); stephanie.dias@mrc.ac.za (S.D.); 2 Department of Obstetrics and Gynaecology, School of Medicine, Faculty of Health Sciences, University of Pretoria, Pretoria 0028, South Africa; sumaiya.adam@up.ac.za; 3 Centre for Cardio-Metabolic Research in Africa (CARMA), Division of Medical Physiology, Faculty of Health Sciences, Stellenbosch University, Tygerberg, Cape Town 7505, South Africa; 4 Diabetes Research Centre, Faculty of Health Sciences, University of Pretoria, Pretoria 0028, South Africa

**Keywords:** gestational diabetes mellitus, gut microbiome, microbiota dysbiosis, fibre, polyphenols

## Abstract

Gestational diabetes mellitus (GDM) is an escalating public health concern due to its association with short- and long-term adverse maternal and child health outcomes. Dysbiosis of microbiota within the gastrointestinal tract has been linked to the development of GDM. Modification of microbiota dysbiosis through dietary adjustments has attracted considerable attention as adjunct strategies to improve metabolic disease. Diets high in fibre and polyphenol content are associated with increased gut microbiota alpha diversity, reduced inflammation and oxidative processes and improved intestinal barrier function. This review explores the potential of fibre and polyphenol supplementation to prevent GDM by investigating their impact on gut microbiota composition and function.

## 1. Introduction

Gestational diabetes mellitus (GDM) is characterized by glucose intolerance that develops during pregnancy and has been identified as the most common metabolic disorder during pregnancy. The condition affects approximately 13.4% of live births globally [1], although the prevalence has been shown to vary according to the diagnostic criteria and population group [2]. The global incidence of GDM has risen rapidly, which has been attributed to the high prevalence of overweight and obesity in females [3]. Apart from significantly increased healthcare costs [4,5], GDM is associated with an increased risk of adverse maternal and neonatal outcomes including cesarean delivery, preterm birth, pre-eclampsia, macrosomia, neonatal hypoglycemia and respiratory distress syndrome [6]. GDM is a transient state of glucose intolerance that typically resolves after birth; however, women with GDM have a seven-fold increased risk of developing type 2 diabetes (T2DM) in later life [7] and a ~four-fold increased risk of developing cardiovascular and coronary artery disease after pregnancy [8]. Offspring born to mothers with GDM have an increased risk for obesity, T2DM, cardiovascular disease and neurodevelopmental disorders compared to offspring of women without GDM [9,10]. Adequate glucose control during pregnancy is critical to mitigate adverse outcomes for mother and child. The first-line treatment for GDM is dietary intervention and physical activity, with these lifestyle modifications able to control blood glucose in the majority of women [11].

The important role of the gastrointestinal tract (GIT) and intestinal microbiota in maintaining health and well-being has been extensively documented [12,13,14], with gut microbiota dysbiosis linked to the development of GDM [15,16,17]. While the exact mechanism linking the microbiome to GDM remains a subject of debate, it is generally accepted that diet is a key determinant in shaping microbiome composition. Food induces rapid changes in microbiota composition [18], which has been associated with metabolic disturbances such as insulin resistance and inflammation [19,20]. Consequently, modulation of microbiome composition through dietary intervention holds therapeutic promise for GDM treatment.

In recent years, the beneficial effects of dietary supplementation on the microbiome have been widely reported. In particular, the health-promoting effects of fibre and polyphenol supplementation have attracted considerable attention as adjunct strategies to improve metabolic disease. Increased consumption of dietary fibre has been demonstrated to promote the growth of short-chain fatty acids (SCFAs)-producing bacteria in the gut, which has been associated with improvements of hyperlipidemia, hyperglycemia, hyperinsulinemia and hypercholesterolemia [21]. On the other hand, polyphenol supplementation has been shown to increase the abundance of health-promoting microbiota such as *Lactobacillus* and *Bifidobacterium*, as well as decrease the abundance of *Clostridium* pathogenic species [22].

This review aims to explore the impact of fibre and polyphenol supplementation on the gut microbiome as a potential therapeutic avenue for GDM. The relationship between GDM and the gut microbiota will be reviewed, followed by discussion on the potential therapeutic strategy of fibre and polyphenol supplementation in modulating the gut microbiome, inflammation and insulin signalling in women with GDM. The review will conclude with an overview of current perspectives and propose directions for future research.

## 2. The Gut Microbiome

The gut microbiome refers to the microbes that colonize the GIT. The human GIT contains an abundant and diverse community of more than 100 trillion microorganisms, which include bacteria, yeast and viruses [23]. Bacteria have been identified as being predominant, with bacterial genes within the GIT outnumbering their host’s genes by more than 100 times. The composition of the gut microbiome is influenced by various factors such as diet, genetics, age, stress, disease and medications [13], contributing to the wide diversity observed among individuals. Studies have consistently documented that the normal gut microbiota comprise two dominant phyla, the gram-positive *Firmicutes* and gram-negative *Bacteroidetes*, accounting for ~90% of the total microbiota. Minor constituents include *Actinobacteria*, *Proteobacteria*, *Fusobacteria*, and *Verrucomicrobia*.

The gut microbiota forms a long-term, dynamically balanced symbiotic relationship with the host, playing a crucial role in metabolism, immune function, the integrity of the gut mucosal barrier, and safeguarding against pathogens [23]. Moreover, gut bacteria have been demonstrated to be integral to the digestive process, facilitating the extraction, synthesis, and absorption of nutrients and metabolites, which encompass bile acids, lipids, amino acids, vitamins, and SCFAs. SCFAs, produced through bacterial fermentation of indigestible nutrients such as dietary fibres and complex carbohydrates, have emerged as key metabolites in the regulation of human metabolism. SCFAs, such as butyrate, propionate, and acetate, can function as energy sources for intestinal epithelial cells while concurrently strengthening the mucosal barrier. In the liver and muscle, SCFAs have been reported to activate 5’ adenosine monophosphate-activated protein kinase (AMPK), which reduces gluconeogenesis in the liver and increases the expression and translocation of glucose transporter 4 (GLUT 4) in the muscle, thereby stimulating glucose uptake [24]. Within adipose tissue, these SCFAs promote adipogenesis, enhance insulin sensitivity, decrease inflammation, and inhibit lipolysis, consequently reducing the presence of circulating free fatty acids (FFAs) [24,25]. The exogenous administration of SCFAs to rodents has demonstrated the induction of thermogenic genes, specifically uncoupling protein 1 (UCP1) and peroxisome proliferator-activated receptor gamma coactivator 1 alpha (PGC1α), in adipose tissue. This process enhances energy expenditure and reduces overall adiposity in the body [25]. In enteroendocrine cells, SCFAs stimulate glucagon-like peptide 1 (GLP-1) expression, which promotes insulin secretion and suppresses glucagon release from the pancreas. This, in turn, contributes to the reduction of body fat and promotes glucose homeostasis [24]. Apart from beneficial effects on lipid and glucose metabolism, SCFAs can inhibit lipopolysaccharides (LPS)-induced inflammation [24] and enhance intestinal permeability by upregulating tight junction proteins, specifically ZO-1 and occludin [26]. Additionally, SCFAs have been observed to upregulate anti-inflammatory regulatory T cells (Treg) in the gut [27] and decrease the levels of pro-inflammatory cytokines, such as interleukin 6 (IL6) and tumor necrosis factor alpha (TNFα) [28].

The advent of next-generation 16S DNA sequencing has allowed the rapid profiling of the gut microbiome, offering valuable insights into its role in health and disease [23]. Compared to bacterial culture, which has proven to be time-consuming and challenging, DNA sequencing allows for the comprehensive characterization of bacterial genomes at the species level, enabling an in-depth taxonomic identification of intricate microbiomes. Utilizing rapid profiling techniques, the presence or absence of specific microbial species has been linked to health or disease conditions. Gut microbiome dysbiosis, which refers to the imbalance between symbiotic and pathogenetic microbiota, has been associated with various metabolic disorders including obesity and T2DM [12,13,14].

## 3. The Gut Microbiome during Pregnancy

Pregnancy induces alterations in the microbiomes of the oral cavity, skin, vagina and gut, with the most pronounced effect observed in the composition of the gut microbiome [29,30,31]. These alterations are thought to occur in response to hormonal, immune system and metabolic changes associated with pregnancy, which contribute to foetal growth and development. In 2012, Koren et al. demonstrated a significant shift in the gut microbiota composition of pregnant women from the first to the third trimesters. The third trimester microbiome was characterized by lower bacterial diversity (α-diversity) and greater inter-individual diversity (β-diversity), which was accompanied by an increase in pro-inflammatory *Proteobacteria* and *Actinobacteria* and a decrease in anti-inflammatory *Faecalibacterium* and butyrate-producing bacteria [32]. These researchers additionally demonstrated that transferring the microbiota from the third trimester to germ-free mice induced insulin resistance, inflammation and weight gain, establishing a causal connection between the microbiome and metabolic disturbances during pregnancy. Similarly, Nuriel-Ohayon et al. reported significant variations in the composition of the gut microbiome between the first and third trimesters [33]. Genera such as *Neisseria*, *Blautia*, *Collinsella* and *Bifidobacterium* were increased, while levels of *Clostridium*, *Dehalobacterium* and *Bacteroidales* were decreased in the third trimester compared to the first trimester.

Using a mouse model, these authors demonstrated that administering progesterone in the third trimester increased *Bifidobacterium* levels, directly implicating pregnancy hormones in influencing the gut microbiome composition [33]. *Bifidobacteria* play a role in regulating weight gain, insulin sensitivity and glucose tolerance, and they have been suggested to be beneficial during pregnancy [34], while reduced levels have been linked to preterm birth [35]. Given that *Bifidobacteria* are lactic acid-producing bacteria that are passed from mother to infant during vaginal birth, increased levels may be an adaptive response to aid milk digestion by infants [33]. Conversely, some studies have reported minimal changes in microbiome composition during pregnancy [36], suggesting that host factors such as diet, weight and antibiotic use may play a significant role in shaping the gut microbiome during pregnancy [31]. Further research is needed to fully understand the role of the gut microbiome during pregnancy.

## 4. The Gut Microbiome during GDM

Pregnancy is associated with major shifts in the gut microbiome, leading to increased gestational inflammation and insulin resistance, potentially contributing to the development of GDM. Accordingly, variations in the composition of the gut microbiome have been extensively documented between healthy pregnancies and those complicated by GDM [15,16,31,37]. The gut microbiome in women with GDM bears similarities to that of adults with T2DM and is characterized by a decrease in α-diversity and an increase in β-diversity [38]. Microbiota dysbiosis during GDM has been extensively reviewed [15,16,31,37] and is beyond the scope of the current review. The exact mechanisms linking microbiota dysbiosis to GDM are still subject to dispute; however, evidence suggests a causal role for the microbiome in the development of GDM [39,40]. These authors showed that first-trimester fecal transplants from women with GDM were capable of inducing GDM in germ-free mice [40]. Furthermore, Dong et al. demonstrated that alterations in fecal microbiota were associated with hyperglycemia-related changes of the plasma metabolome in women with GDM [39]. These studies suggest that therapies to restore gut microbiota dysbiosis could act to alleviate GDM.

Gut microbiota dysbiosis has been reported to contribute to systemic inflammation and the inhibition of insulin signalling [41]. Although the mechanisms that link gut microbiota dysbiosis with insulin resistance remain to be elucidated, systemic inflammation due to “leaky gut” and decreased intestinal barrier function have been suggested as potential triggers. The imbalance characterized by an increase in *Firmicutes* and *Actinobacteria* and a decrease in *Bacteroidetes* disrupts intestinal permeability, leading to increased absorption of LPS, a major component of the outer membrane in Gram-negative bacteria [41]. While it is generally accepted that Gram-negative bacteria are linked to leaky gut, it has been suggested that the endotoxic activity of LPS from the *Bacteroidetes* phylum might be lower than that from other Gram-negative bacteria belonging to the *Proteobacteria* phylum [42]. Moreover, different species within *Bacteroidetes* have been shown to exert distinct metabolic actions [43].

Increased LPS leads to metabolic endotoxemia and the subsequent activation of Toll-like receptor (TLR) 2 and 4 in tissues, including the adipose tissue, muscle and liver. This subsequently leads to the upregulation of inflammatory pathways such as c-Jun NH2-terminal kinase (JNK) and IκB kinase complex (IKKβ)/inhibitor of nuclear factor-κB (IκBα)/nuclear factor-κB (NF-κB). The increased activation of inflammatory pathways leads to impairment of insulin signalling, with decreased phosphorylation of the insulin receptor, insulin receptor substrate (IRS) and Protein kinase B (Akt) [41,44,45]. Liu et al. reported high levels of *Prevotella*, a mucin-degrading bacterium, in women with GDM [46], suggesting that impairment of the gut barrier during GDM may be due to thinning of the mucosal layer overlying the gut epithelium.

SCFAs have been proposed as an additional mechanism linking the gut microbiome to insulin resistance [24]. While SCFAs exert protective effects by strengthening the gut barrier and improving peripheral insulin sensitivity and glucose uptake, the dysregulated and excessive production of SCFAs may also negatively affect insulin sensitivity and disrupt glucose homeostasis, thereby leading to GDM [15]. Furthermore, conflicting results have been reported, with SCFA levels not consistently correlating with beneficial outcomes [47]. Therefore, more research is needed to explore the association between microbiome dysbiosis, SCFAs and metabolic disease.

**Figure 1:**
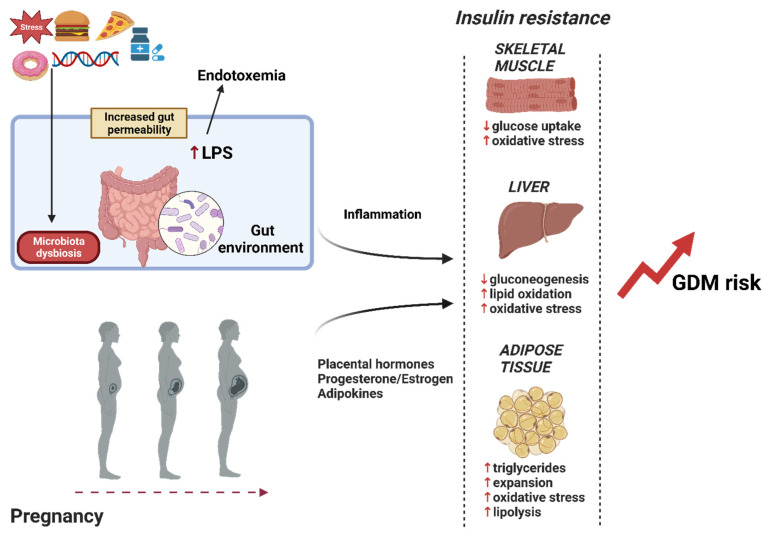
The role of microbiota dysbiosis in the development of GDM. Risk factors such as a high-fat and -sugar diet, genetics, stress and medication alter the gut microbiota composition, leading to dysbiosis. This exacerbates the insulin resistance characteristic of pregnancy and increases GDM risk. Created with BioRender.com. Abbreviations: GDM, gestational diabetes; LPS, lipopolysaccharide.

GDM arises in women who are unable to compensate for insulin resistance that develops during pregnancy [48,49]. Therefore, it is plausible to speculate that gut dysbiosis and its impact on reducing insulin sensitivity may exacerbate pregnancy-induced insulin resistance, thereby increasing GDM risk (Figure 1).

## 5. Targeting the Gut Microbiome to Decrease GDM Risk

The importance of the gut in human disease has been acknowledged as far back as 400 BC when Hippocrates said, “death sits in the intestines” and “bad digestion is the root of all evil” [50]. Targeting the gut microbiome to decrease the risk of disease, including GDM, has become an area of active research. Oral supplementation with probiotics, a blend of live microorganisms introduced into the body for their beneficial qualities, represents a common therapeutic approach to manipulate the gut microbiota as a strategy to prevent or manage GDM. The compliance and acceptability of probiotic capsule intervention among pregnant women appears to be higher than lifestyle interventions, indicating a potential significant advantage in clinical practice [51]. While certain studies have reported the positive impact of probiotics in enhancing glucose metabolism and preventing GDM [52,53,54,55], conflicting results have been reported, with some studies showing no protective effects [54,56,57]. In 2023, a systematic review and meta-analysis of randomized controlled trials (RCTs) that assessed the effects of probiotics on glycemic control and metabolic parameters in women with GDM reported that probiotic supplements improved glycemic control, insulin resistance and lipid profile and reduced neonatal birth weight [55]. However, a recent RCT examining the effect of a mixture of probiotic strains on glycemic parameters in women with GDM, reported that these probiotics did not affect glycemic control, nor did they affect maternal and neonatal outcomes [57]. The discrepancies across studies could be attributed to inter-study heterogeneity due to the bacterial strains used in the probiotic supplement, different dosages and duration of treatment and population, genetic, lifestyle and environmental differences. In addition, gestational age at the time of intervention could influence the protective effects of probiotics. The available studies provide inconclusive results regarding the role of probiotics in preventing GDM, emphasizing the need for further research to ascertain the optimal method of probiotic therapy, taking into account factors such as strain, dosage and timing of intervention in pregnant women. It is noteworthy that current evidence suggests that probiotics may potentially increase the risk of pre-eclampsia [58]. Consequently, additional research is imperative before considering the use of probiotics in pregnant women.

Diet has been recognized as a key determinant shaping the composition of the gut microbiome [19,20]. Consequently, modifying the gut microbiota through dietary adjustments may serve as a therapeutic strategy to prevent or manage GDM. Diets characterized by high fibre content and low saturated fatty acids have been linked to increased gut microbiota diversity, reduced inflammation and lower serum zonulin levels, a marker of intestinal permeability, during pregnancy [59,60]. Additionally, the consumption of polyphenols has been associated with reduced inflammatory and oxidative processes, thereby lowering GDM risk [61]. Therefore, dietary supplementation with fibre and polyphenols may contribute to restoring gut homeostasis, serving as potential strategies for GDM prevention and treatment.

In patients with GDM, nutritional intervention is considered the first line of treatment. However, these dietary recommendations must strike a fine balance, minimizing simple sugar intake while maintaining adequate macronutrient levels and ensuring sufficient dietary fibre [11]. Emphasizing the quality of carbohydrates, by considering their glycemic index, may be more effective in achieving glycemic control compared to a broad restriction of carbohydrates, which could lead to increased fat consumption [62]. Dietary fibre is integral to a healthy diet [63] and, due to its important physiological functions, plays a critical role in diabetes management, particularly in the early stages [64]. This suggests that dietary fibre may be a key contributor to the regulation of glycemia in patients with GDM.

### 5.1. Fibre

Dietary fibres are edible carbohydrate polymers that cannot be digested by human endogenous enzymes [65]. The physicochemical properties, chemical structures and biological activities of dietary fibres have been reviewed extensively [63,65,66,67] and are beyond the scope of this review. Briefly, dietary fibres differ substantially in chemical composition and physicochemical properties, such as solubility and fermentability [63]. Insoluble dietary fibres, which include cellulose and hemi-cellulose, provide “bulk” to the digesta, while soluble fibres such as non-starch polysaccharides, non-digestible or so-called resistant starch, as well as resistant oligosaccharides, may serve as substrates for fermentation by gut microbiota [66,67]. Fermentation of dietary fibre by gut microbiota produce SCFAs, which may beneficially influence host metabolism [68,69,70]. However, soluble fibres also exert direct effects on host metabolism, e.g., by decreasing gastric emptying and increasing transit time through their gel-forming properties, which reduces postprandial glucose spikes and delays nutrient absorption [66,68].

Several studies have investigated fibre supplementation during GDM, with conflicting results. A study published in 1993 showed that fibre supplementation did not improve hyperglycemia in women with GDM [71]; however, more recent studies provide evidence that dietary fibre supplementation can improve symptoms of GDM [72,73,74,75,76,77]. Furthermore, high dietary fibre intake has been associated with a reduced incidence of GDM, while low dietary intake has been associated with an increased risk of GDM [78,79,80,81]. While a study in healthy pregnant women failed to show beneficial effects of a galactooligosaccharide intervention on glucose and lipid metabolism [82], the modification of maternal diets to increase fibre content has been shown to improve glycemic control in women with GDM [83]. Discrepancies between the studies could be attributed to the different types and amount of fibre supplementation and may indicate that fibre supplementation as a therapeutic tool may need to be considered in the context of levels of baseline fibre intake [84]. Notably, the consumption of high-fibre diets by women with GDM has been associated with fewer adverse outcomes such as macrosomia when compared to women consuming a carbohydrate-restricted diet [83]. Zhang et al. reported that dietary fibre supplementation in women with GDM, in combination with nutritional education, lowered the incidence of neonatal hyperbilirubinemia compared to standard nutritional education alone [85]. Despite conflicting results, fibre supplementation during pregnancy has attracted interest as a strategy to prevent the development of GDM, and future studies should dissect different approaches to increasing fibre content in diets, i.e., compare effects of fibre supplements with an overall dietary change to improve macro- and micronutrient availability [86].

**Figure 2:**
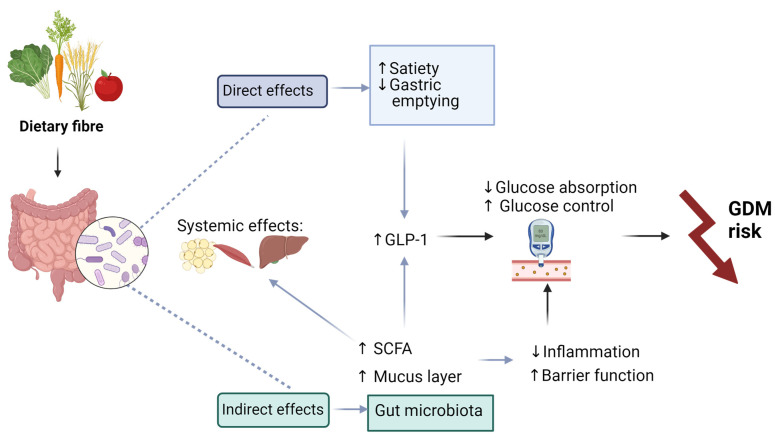
Suggested mechanisms of beneficial effects of dietary fibre supplementation in women with GDM may include direct effects through bulking leading to increased satiety and intestinal transit time. Resultant reduced or slower nutrient absorption may improve glucose homeostasis. Indirect effects of dietary fibre on microbiota composition may improve intestinal barrier function through stimulation of short-chain fatty acids (SCFAs) and mucus production, thereby reducing inflammation and improving insulin sensitivity and thus decreasing GDM risk. SCFAs also exert beneficial effects on tissues responsible for glucose disposal such as adipose and hepatic tissues and skeletal muscle. Created with BioRender.com. Abbreviations: GDM, gestational diabetes; GLP-1, glucagon-like peptide 1, gastrointestinal. SCFA, short-chain fatty acids.

The beneficial effects of fibre against GDM may be mediated by gut microbiota. Several lines of evidence have demonstrated the close relationship between dietary fibre and gut microbiota [19,65,87,88,89] (Table 1). Dietary fibre profoundly and rapidly affects microbiota composition through selection of saccharolytic bacterial strains that digest fibres to produce SCFAs [90,91,92]. SCFAs act to protect the intestinal barrier [93] and possess anti-inflammatory and insulin-sensitizing actions, e.g., through stimulating the release of gastric hormones such as glucagon-like peptide 1 (GLP-1) and peptide YY (PYY) [94]. A study by Röytiö et al. [60] reported an association between fibre and fat intake and microbiota diversity in healthy pregnant women with obesity. In women consuming a high-fibre and moderate-fat diet, the abundance of *Bacteroidaceae* was decreased and fibre intake positively correlated with indices of α-diversity and abundance of the phylum *Firmicutes* [60]. Another study showed that a low-fibre diet was associated with the overgrowth of *Collinsella* spp. and hyperinsulinemia in pregnant women with obesity or overweight [95]. Furthermore, in healthy women, supplementation with galactooligosaccharides during pregnancy increased the abundance of *Paraprevotella* and *Dorea*, whereas the abundance of *Lachnospiraceae* was decreased, although these changes did not affect the incidence of GDM [82]. While these studies show that dietary fibre affects microbiota composition and metabolic responses in pregnancy, a limited number of studies have investigated the effect of dietary fibre supplementation on microbiota composition and diversity in women with GDM. One study found that women with GDM displayed an increased abundance of *Collinsella*, which was correlated with increased insulin levels in these women [96]. Using a mouse model of GDM, Miao et al. [97] showed that feeding insulin resistant and dyslipidemia pregnant mice with inulin-type fructans improved insulin sensitivity and reduced triglyceride and low-density lipoprotein (LDL) cholesterol levels while increasing microbiota richness and diversity. At the genus level, treatment with inulin-type fructans increased the abundance of *Bifidobacterium* and *Verrucomicrobia* (specifically *Akkermansia muciniphila*), while the abundance of *Dubosiella* was decreased. Sugino et al. [98] showed that a diet high in complex carbohydrates improved microbiota composition in mothers and infants and could therefore be regarded as a viable diet choice for women with GDM. The study showed that the dietary intervention increased the abundance of *Bifidobacteriaceae*, which has been suggested to possess beneficial effects during pregnancy [34]. We propose that the direct effects of dietary fibre on the small intestine, the production of microbial SCFA and the increase in mucin-degrading bacterial strains may contribute to the improvement of GDM symptoms (Figure 2).

While the existing literature on fibre supplementation contains inconsistencies, possibly attributed to the variety of dietary fibres with distinct chemical compositions and physiological properties [96,99], it is evident that fibre supplementation could be beneficial for treatment of GDM. However, current evidence for associations between microbiota composition and fibre intake in women at risk of developing GDM or women diagnosed with GDM stems predominantly from observational studies (summarized in Table 1), while intervention trials examining microbiota composition are scarce. Furthermore, a recent study reported that dietary fibre was unable to improve intestinal barrier dysfunction or alter microbiota composition in women who developed hypertension during pregnancy [100]. Therefore, intervention with dietary fibre supplements to improve GDM and prevent adverse pregnancy outcomes in mothers and children will likely require individualized approaches. These individual approaches could include the analysis of microbiota composition and diversity in individual patients as a guide to choose which fibre supplementation would be best suited. Furthermore, focusing on holistically improving diet rather than using isolated fibre supplements needs to be evaluated in future research. Improving dietary fibre intake through increasing fruit and vegetables will concurrently increase intake of other beneficial dietary components such as polyphenols that may work synergistically with fibre to improve metabolic health [68].

### 5.2. Polyphenols

Polyphenols, natural compounds derived from plants, are gaining increasing recognition for their positive impact on health and their potential in treating metabolic disorders. Predominantly found in fruits, vegetables, and herbal teas, polyphenols encompass various classes such as phenolic acids, phenolic amides, and flavonoids, each characterized by distinct chemical structures and complexities. Polyphenols have been demonstrated to exert therapeutic potential against obesity, T2DM, cardiovascular disease, cancer and neurodegenerative diseases [101,102,103,104]. These beneficial effects are attributed to the antioxidant and anti-inflammatory properties inherent in polyphenols.

The evidence on the ability of polyphenols to reduce the risk of GDM remains inconclusive. Pham et al. reviewed ten studies, of which, five showed a significant, inverse association between the intake of polyphenol-rich foods and GDM, five studies reported no association, and two showed a positive association [105]. Another systematic review, which evaluated eight articles, found no association between polyphenol consumption and GDM [106]. In contrast, a systematic review of 14 studies identified an association between polyphenol intake and reduced GDM risk, which was attributed to the attenuation of inflammatory and oxidative processes [61]. Taken together, these studies suggest a potential correlation between polyphenol consumption and reduced GDM risk, although further research may be required to confirm this association.

A complex and reciprocal interplay between polyphenols and gut microbiota exist, with polyphenols believed to exert their advantageous health effects by influencing the composition of the gut microbiota. Polyphenols exert prebiotic-like effects, promoting the growth of beneficial bacteria and inhibiting pathogenic bacteria, thereby contributing to overall gut health. Extracts rich in polyphenols, derived from sources like cranberries [107], grapes [108], pomegranates [109], red pitaya fruit [110], caffeic acid [111], rhubarb [112] and lingonberries [113] have been documented to stimulate the growth of *Akkermansia muciniphila* in the gut of animal models, and in human gut microbiota. Decreased levels of *A. muciniphila* have been associated with dysregulated adipose tissue metabolism, metabolic disorders, and inflammation in obese mice, while increased levels have demonstrated beneficial effects against obesity, T2DM, and gut inflammation [114]. Conversely, certain polyphenol extracts, such as flaxseed extract, have been shown to decrease *A. muciniphila* levels [115]. These contradictory findings underscore the heterogeneity in polyphenol composition and the inherent challenges in characterizing microbiome composition, which is influenced by various factors, including disease state and demographic factors such as age, sex and body mass index [116]. Yuan et al. demonstrated that green tea polyphenols increased the *Firmicutes* to *Bacteroidetes* ratio and elevated SCFA producing genera including *Faecalibacterium*, *Blautia*, *Bifidobacterium*, *Roseburia*, *Eubacterium* and *Coprococcus* [116]. Two-month daily intake of orange juice, rich in flavanones like hesperidin and naringin, resulted in an increased population of *Bifidobacterium* spp. and *Lactiplantibacillus* spp., accompanied by enhanced SCFA production in healthy women, which correlated with an improved metabolic profile and insulin sensitivity [117].

A combination of the flavonoid quercetin and the stilbene resveratrol was reported to protect against diet-induced obesity by inhibiting weight gain, visceral adiposity, serum lipids and inflammatory markers, which was associated with microbiome modulation [118]. These effects were suggested to be attributed to restoration of gut microbiota dysbiosis (decreased *Firmicutes*/*Bacteroides* ratio and increased *Bacteroidales*, *Christensenellaceae*, *Akkermansia* and *Ruminococcaceae*. The xanthone mangiferin exhibited anti-inflammatory and cholesterol-lowering effects in a mouse model of atherosclerosis, which was associated with increased beneficial taxa *Akkermansia*, *Parabacteroides*, and *Bifidobacteriaceae*, and reduction of the pathogenic genus *Helicobacter* [119]. The studies highlighted above suggest that the beneficial health effects of polyphenols occur by modulating gut microbiota (Table 2). The effect of polyphenols on microbiota composition has been extensively reviewed and is beyond the scope of this study [50,120,121]. Taken together, these results indicate that dietary polyphenols possess prebiotic properties and can potentially restore dysregulated gut microbiota, although the precise mechanisms necessitate further elucidation.

Apart from modulating microbiome composition, polyphenols may confer additional benefits to the host. Only 5–10% of ingested polyphenols are absorbed in the small intestine, where they regulate crucial aspects such as (1) mucous and antimicrobial peptide secretion, (2) barrier function, (3) immunoglobulin and cytokine secretion and (4) molecular signalling pathways [120]. Due to their complex structures and high molecular weights, the majority of polyphenols (90–95%) are absorbed in the large intestine. Here, they are metabolized by gut microbiota, increasing their bioavailability and facilitating their bioactive effects. Colonic microbiota, therefore, play an essential role in the breakdown of polyphenolic structures into metabolites that can be easily absorbed to mediate beneficial health effects. The health effects of polyphenol gut microbiota metabolites in different animal models, and in vitro human cell assays have been widely documented [50,114,121], although evidence supporting their bioactivity in humans is limited. This is thought to be mainly attributed to interindividual variability in the gut microbiome, which varies by population and dietary habits.

**Figure 3:**
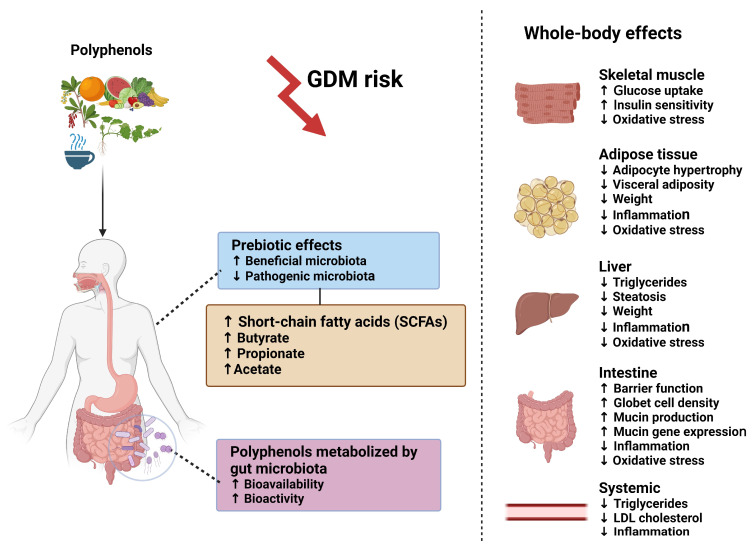
The health benefits of dietary polyphenol supplementation on GDM risk may be mediated by the gut microbiota, decreasing inflammation, oxidative stress, adiposity and dyslipidemia, thereby improving insulin sensitivity and glucose uptake. Created with BioRender.com. Abbreviations: GDM, gestational diabetes; LDL, low-density lipoprotein.

The potential health benefits of dietary polyphenol supplementation on GDM risk may be mediated by the gut microbiota through prebiotic effects, increasing beneficial microbiota, as well as SCFAs such as butyrate, propionate and acetate. Alternatively, gut microbiota may metabolize polyphenols, enhancing their bioavailability and bioactivity. Beneficial whole-body effects include increased glucose uptake and insulin sensitivity; decreased oxidative stress in skeletal muscle; decreased adipocyte hypertrophy, visceral adiposity, weight, inflammation and oxidative stress in adipose tissue; decreased triglycerides, steatosis, weight, inflammation and oxidative stress in the liver; improved barrier function, goblet cells, mucin production and gene expression, decreased inflammation and oxidative stress in the intestine; and decreased systemic triglycerides, inflammation and LDL cholesterol, thereby decreasing GDM risk (Figure 3).

Although it is generally accepted that polyphenols are antioxidants, evidence suggests that they may exhibit pro-oxidant properties when in excess [122,123]. Therefore, it is important to consider the risks associated with polyphenol consumption during pregnancy, and further studies are needed to investigate the safety of polyphenol consumption [124]. More research is required to determine the optimal dose of polyphenols, or to observe the potential side effects of long-term exposure in humans.

## 6. Limitations

Fibre and polyphenol supplementation offer potential health benefits through modulation of the gut microbiome in women with GDM. However, understanding the complex interplay between these dietary supplements, the microbiome and health faces several challenges. One of the key challenges is the substantial individual variation in microbiome composition, influenced by factors such as genetics, diet, demographics and lifestyle [125]. Furthermore, this review highlights the scarcity of human studies investigating the interplay between the microbiome and fibre or polyphenol supplementation, while those that are conducted are limited by small sample sizes [109,116,117]. The randomized controlled trials using fibre interventions have often lacked a suitable placebo control, thus preventing blinding, and increasing risk of bias [72,73,74,75,77,85]. Other studies have focused on specific outcomes other than effects of fibre on GDM symptoms or risk [98], or used other fibres as placebo [82], and therefore did not show clear beneficial effects of fibre on GDM symptoms or risk. There is a need for well-designed randomized control human studies that consider different populations, varying doses and durations of fibre or polyphenol intake. Future research should account for the dynamic nature of microbiota [126] and assess the temporal dynamics of microbiome and fibre or polyphenol interactions. Additionally, the complexity of fibre and polyphenols, including diverse structures and bioavailability [127], underscores the challenge of generalizing findings across all fibre supplements and polyphenolic compounds. The molecular mechanisms underlying the interaction between the microbiome and fibre or polyphenols remain unclear, which is partly attributed to the challenges highlighted above. Overcoming these limitations will contribute to a better understanding of the intricate relationship between diet, the microbiome and human health and advance microbiome, fibre and polyphenol studies toward clinical translation.

## 7. Future Perspectives

- Accumulating studies have highlighted potential links between the composition of the gut microbiome and the development of GDM; however, the existing evidence remains inconclusive [15,16,31,37]. Further research is warranted to delineate the gut microbiome profiles in both normal pregnancies and those complicated by GDM. These studies should be conducted in diverse geographical populations, ensuring an adequate sample size to account for lifestyle-associated factors such as diet, physical activity and antibiotic use. Additionally, it is crucial to consider technological factors such as sample processing and sequencing platform, as they are known to influence the identification of bacterial taxa in the GIT [42].
- As outlined in this review, additional investigation is necessary to explore the potential of dietary supplementation with fibre and polyphenols to shape the maternal microbiome as a nutritional intervention strategy in women with GDM. The response to food intake is influenced by genetic, epigenetic and microbial factors, underscoring the need for a patient-centred, personalized approach in the nutritional therapy of GDM [125]. RCTs to evaluate the effect of dietary modulation on shaping the gut microbiota during pregnancy and its potential to prevent or control GDM are required.
- The mother’s microbiota has the potential for vertical transmission to the offspring. Further investigations are essential to explore the influence of the maternal microbiome on foetal programming and understand how the infant microbiome might affect the physiology and long-term health of newborns [17,29,31].
- Microbiota in different locations, such as the gut, oral cavity and vagina, have been linked to GDM [38,128,129]. For example, a dysbiotic vaginal microbiome is associated with increased inflammatory cytokine expression, while increased periodontal bacteria in the oral microbiome is associated with GDM risk. Therefore, integrative studies across these body sites are required to provide a better understanding of microbial crosstalk during GDM.
- The maternal microbiome plays an important role in producing metabolites that impact both health and disease. Throughout pregnancy, the gut microbiota undergoes profound changes, resulting in an increase of pathogenic lactic acid-producing bacteria and a reduction in beneficial butyrate-producing bacteria [29]. Research to examine the relationship between metabolomics and microbial diversity is required.
- Dysbiosis in gut microbiota may serve as a potential indicator for the development of T2DM post-pregnancy [130], suggesting that modifying the gut microbiota through dietary interventions could improve diabetes-related outcomes. Subsequent research should investigate ways to ameliorate gut bacterial dysbiosis and assess the effectiveness of potential interventions, including supplementation with fibre and polyphenols, particularly among pregnant women.

## 8. Conclusions

The maternal gut microbiome modulates metabolic health and affects insulin resistance during pregnancy, playing an important role in the development of GDM. Additionally, gut microbiota dysbiosis has been associated with adverse pregnancy outcomes [17,29,31] and identified as a potential predictor of T2DM [130]. Therefore, it is crucial to conduct studies examining whether dietary interventions such as fibre and polyphenol supplementation can ameliorate gut microbial dysbiosis, ultimately improving outcomes related to GDM. These investigations should encompass diverse populations of pregnant women, considering factors like gestational age, body weight and medication use, as these variables are known to influence the maternal microbiome.

## Figures and Tables

**Table 1:** Effect of fibre consumption on microbiota composition and metabolic parameters.

Reference	Fibre	Study Design	Effect on Gut Microbiota	Study Findings
Schroeder et al., 2018 [89]	Inulin	Male C57BL/J6 mice were fed a western style diet (WSD) and 1% inulin or *Bifidobacterium longum* or a combination of inulin and *B. longum* for 4 weeks.	Effects on microbiota composition were not measured.	None of the treatments affected metabolic parameters, however, both treatments improved mucus function in WDS-fed mice. Inulin treatment prevented the penetrability of the intestinal mucus layer and *B. longum* treatment restored mucus growth.
Zou et al., 2018 [88]	Inulin	Male C57BL/6 mice were fed a high fat diet (HFD) containing 20% cellulose (non-digestible fibre control) or HFD containing 20% inulin fibre (soluble fibre) for 4 weeks. Gut microbiota was assessed by 16S rRNA sequencing of fecal samples.	Inulin treatment increased gut bacterial load and the abundance of *Bifidobacteriaceae*. Inulin reduced the *Firmicutes/Bacteroidetes* ratio and the abundance of *Proteobacteria*, *Streptococcus*, *Clostridium*, and *Enterococcaceae*.	Inulin reduced body weight gain, dysglycemia, hepatic steatosis and adiposity (adipocyte size). Treatment had no effect on serum triglycerides but decreased cholesterol levels were reported.
Song et al., 2019 [70]	Inulin	Male C57BL/6J mice and *ob/ob* mice were fed a chow diet supplemented with inulin (10 g/kg body weight/day) in the drinking water for 4 weeks. Gut microbiota was assessed by 16S rRNA sequencing of cecal samples.	Inulin supplementation reduced α-diversity and decreased the *Firmicutes/Bacteroidetes* ratio. Inulin increased the abundance of *Prevotellaceae UCG 001*, *Oscillibacter*, *Lachnospiraceae UCG 006*, *Lachnospiraceae UCG 008*, *Enterobacter*, and *Parvibacter*.	Inulin supplementation reduced food intake and total cholesterol and improved glucose tolerance and liver steatosis. Improvement in metabolic parameters were associated with increased production of SCFAs by *Prevotellaceae UCG 001*.
Miao et al., 2022 [97]	Inulin-type fructan (ITF)	Female C57BL/6J mice were fed a high fat/high sucrose (HFHS) diet for 4 weeks prior to pregnancy and for 18 days during pregnancy. Mice received 3.33 g/kg bodyweight ITF per day by oral gavage for the duration of HFHS feeding. Gut microbiota was assessed by 16S rRNA sequencing of fecal samples.	ITF increased α-diversity and fecal SCFAs (butyrate and acetate) production. ITF further increased the abundance of *Verrucomicrobia*, *Bifidobacterium* and *Akkermansia*, while levels of *Dubosiella* were reduced.	ITF treatment reduced body weight gain and improved glucose tolerance and lipid metabolism (decreased triglycerides, total and low density lipoprotein (LDL) cholesterol) in HFHS-fed mice, which was asscoaited with increased SCFA modulating gut microbiota.
Paone et al., 2022 [87]	Fructo-oligosaccharides (FOS)	Male C57BL/6 J mice were fed a HFD with 10% FOS in the drinking water for 6 weeks. Gut microbiota was assessed by 16S rRNA sequencing of fecal samples.	FOS treatment increased the abundance of *Odoribacter*, *Akkermansia*, *Muribaculaceae* and *Ruminococcaceae*.	FOS treatment reduced body weight and improved glucose tolerance in HFD-fed mice. FOS increased plasma glucagon-like peptide 1 (GLP-1) levels and the number of intestinal goblet cells producing mucins. The genera increased by FOS treatment correlated negatively with glucose tolerance but were positively associated with mucus layer/mucus production.
Röytiö et al., 2017 [60]	No intervention	100 women with overweight/obesity were enrolled at <17 weeks of gestation. Three-day food records were collected before the study. Gut microbiota was assessed by 16S rRNA sequencing of fecal samples.	The recommended fibre and fat intake correlated with increased microbiota richness and α-diversity.	Microbiota diversity and richness inversely correlated with inflammatory markers.
Ferrocino et al., 2018 [96]	Standard nutritional recommendations	41 patients with GDM were enrolled between 24 and 28 weeks of gestation. A dietary questionnaire was conducted. Gut microbiota was assessed by 16S rRNA sequencing of fecal samples.	α-diversity significantly increased in women with GDM. The abundance of *Firmicutes* increased and abundance of *Bacteroidetes* and *Actinobacteria* decreased.	Adherence to nutritional recommendations decreased the abundance of *Bacteroides. Faecalibacterium* correlated with fasting glucose concentrations. *Collinsella* was positively and *Blautia* inversely correlated with insulin and HOMA-IR. *Sutterella* was associated with inflammatory marker C-reactive protein.
Gomez-Arango et al., 2018 [95]	No intervention	57 women with overweight and 73 women with obesity were enrolled at 16 weeks of gestation. A dietary questionnaire was used to assess macronutrient intake in these women. Gut microbiota was assessed by 16S rRNA sequencing of fecal samples.	Low fibre intake correlated with increased levels of *Collinsella* and lactate producing bacteria. High fibre intake was associated with SCFA producing bacteria.	Low fibre intake may allow overgrowth of *Collinsella*, which was correlated with increased insulin levels.
Sugino et al., 2022 [98]	Meals with different types (same amount) of fibre provided	34 women with GDM were randomised to either CHOICE diet (60% complex carbohydrates, 25% fat) or conventional diet (40% complex carbohydrates, 45% fat) from 30 weeks of gestation. Gut microbiota was assessed by shotgun metagenomic sequencing of fecal samples.	CHOICE diet increased the abundance of *Bifidobacteriaceae* (*B. adolescentis*) in women with GDM.	Maternal glucose levels did not differ between the treatment groups. The CHOICE diet increased infant α-diversity over time.
Wan et al., 2023 [82]	Galacto-oligosaccharides (GOS), 60 g per day	52 women were enrolled at 6–8 weeks of gestation with follow up at 11–13 weeks and 24–28 weeks. Women received 60 g of GOS daily. Gut microbiota was assessed by 16S rRNA sequencing of fecal samples.	GOS increased the abundance of *Paraprevotella* and *Dorea* spp. and decreased the abundance of *LachnospiraceaeUCG_001*.	No differences in GDM incidence, fasting plasma glucose, lipids, interleukin 6 (IL-6) and neonatal outcomes were observed.

**Table 2:** Effect of dietary polyphenols on microbiota composition and metabolic parameters.

Reference	Polyphenol	Study Design	Effect on Gut Microbiota	Study Findings
Anhé et al., 2015 [107]	Cranberry extract	High-fat/high-sucrose (HFHS)-fed male C57BL/6J mice were treated with 200 mg/kg of cranberry extract daily for 8 weeks. Gut microbiota was assessed by 16S rRNA sequencing of fecal samples.	The cranberry extract increased levels of the mucin-degrading bacterium *Akkermansia*.	The cranberry extract protects against diet-induced obesity (decreased weight gain, visceral obesity, liver weight and triglyceride accumulation), insulin resistance and intestinal inflammation in association with increased *Akkermansia* spp. in the gut microbiota of HFHS-fed mice.
Roopchand et al., 2015 [108]	Grape extract	Male C57BL/6J mice were fed a high-fat diet (HFD) containing 1% Concord grape polyphenols for 13 weeks. Gut microbiota was assessed by 16S rRNA sequencing of cecal and fecal samples.	The grape extract increased levels of *Akkermansia muciniphila* and decreased the ratio of *Firmicutes* to *Bacteroidetes*.	The grape extract improved metabolic outcomes (weight gain and adiposity) and lowered intestinal and systemic inflammation in association with increased *Akkermansia* spp. in the gut microbiota of HFD-fed mice.
Heyman-Lindén et al., 2016 [113]	Lingonberries extract	Male C57BL/6J were fed HFD diet with 20% lingonberries for 11 weeks. Gut microbiota was assessed by 16S rRNA sequencing of cecal samples.	The lingonberries extract increased the abundance of *Akkermansia* and *Faecalibacterium*.	The lingonberries extract was able to prevent diet-induced low-grade inflammation, which was associated with an increase in *Akkermansia* and *Faecalibacterium*.
Power et al., 2016 [115]	Flaxseed	Male C57BL/6 male mice were fed a maintenance diet supplemented with 10% flaxseed for 3 weeks. Gut microbiota was assessed by 16S rRNA sequencing of fecal samples.	The flaxseed extract increased *Prevotella* spp. and reduced *Akkermansia muciniphila* abundance.	The flaxseed extract exhibited beneficial responses contributing to an enhanced mucus barrier (increased goblet cell density, mucin production, and mucin gene expression), which was associated with a 20-fold increase in *Prevotella* spp. and a 30-fold reduction in *Akkermansia muciniphila* abundance.
Song et al., 2016 [110]	Red pitaya fruit extract	Male C57BL/6J mice were fed a HFD containing 200 mg/kg red pitaya extract for 14 weeks. Gut microbiota was assessed by 16S rRNA sequencing of fecal samples.	The red pitaya fruit extract decreased *Firmicutes* and increased *Bacteroidetes* and *Akkermansia*.	The red pitaya extract protects against diet-induced obesity and its related metabolic disorders (reduced weight gain, visceral adiposity, improved hepatic steatosis, adipose hypertrophy, insulin resistance and inflammatory status) by decreasing the ratio of *Firmicutes* and *Bacteroidetes* and increasing *Akkermansia* in the gut microflora.
Zhang et al., 2016 [111]	Caffeic acid	Female C57BL/6 mice with colitis were fed a diet with 1 mM caffeic acid. Gut microbiota was assessed by 16S rRNA sequencing of fecal samples.	Caffeic acid decreased *Firmicutes* and increased *Bacteroidetes* and the mucin-degrading bacterium *Akkermansia*.	The caffeic acid exerted anti-inflammatory effects which was associated with a decrease in the *Firmicutes*/*Bacteroidetes* ratio and an increase in *Akkermansia* in mice with colitis.
Neyrinck et al., 2017 [112]	Rhubarb extract	Male C57BL/6J mice were fed a control diet supplemented with 0.3% Rhubarb extract for 17 days and thereafter challenged with 30% *w*/*v*, 6 g/kg body weight alcohol. Gut microbiota was assessed by 16S rRNA sequencing of cecal samples.	The rhubarb extract increased *Akkermansia muciniphila* and *Parabacteroides goldsteinii*.	The rhubarb extract improved alcohol-induced hepatic injury and downregulated markers of inflammation and oxidative stress in the liver, which was associated with increased *Akkermansia muciniphila* and *Parabacteroides goldsteinii*.
Zhao et al., 2017 [118]	Resveratrol and quercetin	Male Wistar rats were fed a HFD diet with a combination of quercetin (30 mg/kg body weight) and resveratrol (15 mg/kg body weight) daily for 10 weeks. Gut microbiota was assessed by 16S rRNA sequencing of fecal samples.	Resveratrol and quercetin decreased *Firmicutes* and increased *Bacteroidales*, *Christensenellaceae*, *Akkermansia* and *Ruminococcaceae*. Levels of *Desulfovibrionaceae*, *Acidaminococcaceae*, *Coriobacteriaceae*, *Bilophila* and *Lachnospiraceae* were decreased.	Resveratrol and quercetin reduced HFD-induced weight gain, visceral adiposity, serum lipids and inflammatory markers, which was associated with microbiome modulation.
He et al., 2023 [119]	Mangiferin	Female ApoE−/− mice were fed a high-choline diet plus 0.5% mangiferin for 15 weeks. Gut microbiota was assessed by 16S rRNA sequencing of fecal samples.	Mangiferin increased beneficial taxa *Akkermansia*, *Parabacteroides* and *Bifidobacteriaceae* while reducing the pathogenic genus *Helicobacter*.	Mangiferin exhibited anti-inflammatory and cholesterol-lowering effects, which was associated with microbiome modulation.
Li et al., 2015 [109]	Pomegranate extract	20 healthy volunteers (9 females and 11 males) received 1000 mg of pomegranate extract daily for 4 weeks. Gut microbiota was assessed by 16S rRNA sequencing of fecal samples.	The pomegranate extract increased levels of *Akkermansia muciniphila* and *Proteobacteria* and decreased *Actinobacteria*.	The article did not assess the health benefits of the pomegranate extract, but suggests that it may mediate beneficial effects on weight maintenance and insulin sensitivity by changing the ratio of *Firmicutes* to *Bacteroidetes* and increasing *Akkermansia* in the gut microflora.
Yuan et al., 2018 [116]	Green Tea	12 healthy volunteers (4 females and 8 males) received 400 mL of green tea liquid daily for 2 weeks. Gut microbiota was assessed by 16S rRNA sequencing of fecal samples.	Green tea increased the *Firmicutes* to *Bacteroidetes* ratio and elevated short chain fatty acid (SCFA) producing genera *Faecalibacterium*, *Blautia*, *Bifidobacterium*, *Roseburia*, *Eubacterium* and *Coprococcus*.	The green tea increased SCFA-producing bacteria and reduced the expression of functional markers of inflammation (lipopolysaccharide (LPS) synthesis). The overall composition of the gut microbiota was influenced by age, sex, body mass index and the status of bowel movements (however, these factors did not influence baseline or green tea intervention microbiome profile).
Lima et al., 2019 [117]	Orange juice	10 healthy females received 300 mL of orange juice daily for of 60 days. Gut microbiota was assessed using bacterial culture and polymerase chain denaturing gradient gel electrophoresis (DGGE) of fecal samples.	Orange juice increased the population of *Bifidobacterium* spp. and *Lactobacillus* spp.	Daily intake of orange juice improved blood biochemical parameters, such as low-density lipoprotein-cholesterol, triglycerides, glucose and insulin sensitivity, which was associated with increased *Bifidobacterium* spp. and *Lactobacillus* spp.

## Data Availability

No new data were created.
